# Prevalence and determinants of post-traumatic stress disorder five months after the 2019 huge flooding in Iran

**DOI:** 10.1186/s12889-024-17861-y

**Published:** 2024-02-01

**Authors:** Amir Shabani, Maryam Rasoulian, Morteza Naserbakht, Mitra Hakim Shooshtari, Ahmad Hajebi, Amir Tiyuri, Seyed Abbas Motevalian

**Affiliations:** 1 Mental Health Research Center, Psychosocial Health Research Institute, Iran University of Medical Sciences, Tehran, Iran; 2 Department of Psychiatry, School of Medicine, Iran University of Medical Sciences, Tehran, Iran; 3 Research Center for Addiction and Risky Behaviors (ReCARB), Psychosocial Health Research Institute (PHRI), Iran University of Medical Sciences, Tehran, Iran; 4 Department of Epidemiology, School of Public Health, Iran University of Medical Sciences, Tehran, Iran

**Keywords:** Post-traumatic stress disorder, Floods, Disaster, Prevalence, Screening, Epidemiology

## Abstract

**Background:**

Despite the high occurrence of floods in Iran, its psychological consequences have been less discussed. The present paper addresses the prevalence of Post-traumatic Stress Disorder (PTSD) and its determinants among the affected adults by the huge flood of 2019.

**Methods:**

An analytical cross-sectional study was conducted through household face-to-face surveys in August and September 2019. Individuals who were affected by floods and were at least 16 years old were randomly selected from three provinces in Iran: Lorestan and Khuzestan in the west and southwest, and Golestan in the northeast. The questionnaire of demographic and flood related variables in addition to the Impact of Event Scale-Revised (IES-R) were utilized to collect the data. We applied a complex sample analysis to describe the prevalence of PTSD and logistic regression analyses to find its determinants.

**Results:**

Out of the 2,305 individuals approached for surveys, 1,671 (72.5%) adults affected by the floods participated in the study. The majority of participants were housewives, married, had either no formal education or primary education, and resided in rural areas. The prevalence of PTSD in the participants was 24.8% (CI 95%: 20.7–28.8%) and was significantly higher in Lorestan province (39.7%, *P* < 0.001). Determinants of PTSD, were unemployment (adjusted odds ratio [AOR] = 3.53, CI 95%: 1.38-9.00), primary (AOR = 2.44, CI 95%: 1.10–5.41) or high school (AOR = 2.35, CI 95%: 1.25–4.40) education (vs. university), a history of mental disorders (AOR = 2.36, CI 95%: 1.22–4.58), high damage to assets (AOR = 2.29, CI 95%: 1.40–3.75), limited access to health care services after the flood (AOR = 1.95, CI 95%: 1.20–3.19), not receiving compensation for flood damage (AOR = 1.94, CI 95%: 1.01–3.83), high wealth index (AOR = 1.90, CI 95%: 1.23–2.93), and flooded house with a height of more than one meter (AOR = 1.66, CI 95%: 1.02–2.76).

**Conclusion:**

Results show a notable prevalence of PTSD, especially in Lorestan province, among adults affected by floods. Determinants of PTSD include unemployment, lower education, psychiatric history, extensive property damage, limited post-flood healthcare access, lack of compensation, and increased flood exposure. We recommend adopting an inclusive screening approach for high-risk groups and developing appropriate therapeutic and supportive interventions.

## Introduction

Some Studies have demonstrated that over the past five decades, coinciding with the escalation of global warming, floods have emerged as the most prevalent natural disaster [1, 2]. In Iran, more than 80% of the cities are exposed to floods and the incidence of floods has been increasing in recent decades [3, 4]. The flood damage is very extensive and is economically, socially and healthily significant [1, 5].

In 2019, Iran experienced a huge flood due to heavy rains occurring from March to April, primarily impacting the northeastern, western, and southwestern regions [6]. The flood affected thirty-one provinces, 3,800 towns and villages, and 140 rivers. It resulted in the destruction of 65,000 houses, damage to 159 main roads, 700 bridges, and a disruption to the Health System Response [7, 8].

Floods can cause a wide range of health impacts, including immediate or short-term physical, infectious, chemical, family, and residential consequences [9]. They can also lead to reduced access to medical and mental health services, as well as disruptions in mental health [10]. According to the studies, among the psychological consequences of floods, post-traumatic stress and depressive disorders were the most frequent [11, 12].

Studies indicate that the psychological consequences of flooding are more prominent in the first months [11], but these problems are not limited to the immediate aftermath of flooding and can continue to be significant in the following months and even years. For example, in Australia, Matthews et al. [13] examined the mental health status of the flooded population in a cross-sectional study six months after the Northern New South Wales flood. At this point in time, more than a fifth of the population was still distressed about the flood. As another example, we can mention the high frequency of Post-traumatic Stress Disorder (PTSD) and depression in the flooded agricultural population in Korea, even 18 months after the flood [14]. A study on the victims of the spring 2019 floods in Iran revealed that six months after the event, the prevalence of psychological distress and depression was 33.6% and 23.0%, respectively [15]. In the study on PTSD after the flood in Iran, research conducted in Mazandaran Province [16] three months after the 2012 flood reported a PTSD prevalence of 64%, while a recent study in Lorestan province [17] reported it to be 12.8% one year after the 2019 flood.

Despite the high frequency of floods and the extensive material damage they cause in Iran, there are few references, and a substantial gap still exists in understanding the psychological consequences of floods in the Iranian context. After the huge and extensive flooding in Iran in 2019, there arose a necessity to run projects to answer the questions on the flood’s consequences. In line with the President’s decision to establish a “Special Commission for National Report on Floods” with the aim of providing scientific, credible and accurate answers to the various aspects of the floods, the Mental Health Committee was formed as a subset of the Social, Cultural and Media working group. The committee’s studies used a combined approach of qualitative and quantitative research methods, including interviews, group discussions, observations, and a wide-ranging household study, to comprehensively explore the mental health in flood-affected provinces. The present paper addresses the prevalence of PTSD and its determinants among adults affected by the huge flood in 2019, as part of a larger study [15].

## Methods

### Study design and sampling

An analytical cross-sectional study was conducted through household face-to-face surveys in August and September 2019, using three-stage random sampling. The present report focuses on the prevalence of PTSD and its determinants among the flooded adults, as part of a larger study [15]. The individuals affected by the flood, aged at least 16 years, were randomly selected from three heavily affected provinces: Lorestan [including Pol Dokhtar and Mamulan], Khuzestan [including Dasht-e-Azadegan, west Ahvaz and Hamidiyeh] in the west and southwest, and Golestan [including Aq Qala and Gomishan] in the northeast of Iran. The study utilized a three-stage random sampling method (detailed in another paper [15]), with 210 clusters, each comprising 8 adult participants. In the first stage, 70 clusters were selected in each of three provinces based on the ratio of flood victims in cities and villages. This involved identifying affected areas on a map and randomly choosing blocks. In the second stage, households were selected within each block by choosing one plaque randomly and including eight households every second plaque. In the third stage, one adult from each household was randomly chosen based on the proximity of their birthdate to the survey day.

### Criteria for study exclusion

Individuals who were absent from home until the end of data collection, lacked an understanding of the Persian language, were aged less than 16 years, or had physical or cognitive impairments preventing participation in the surveys were excluded from the study.

### Data collection and quality assurance

The data collectors included 18 mental health experts, with 6 assigned to each province to complete the questionnaires on behalf of participants after conducting the surveys. They held a bachelor’s or master’s degree in psychology or related disciplines and underwent a 16-hour hands-on workshop to learn how to implement the protocol and collect data using reliable and widely accepted standardized questionnaires. An observer was assigned for each province to ensure the accurate selection of the sample and to review the completed forms for precision and accuracy.

### Instruments

#### 1) A questionnaire of demographic variables

including age, gender, marital and employment status, education, being urban or rural (as the place of residence), and the ownership of assets (in detail in another paper [15]), was administered. Additionally, participants self-reported their past history of mental disorders diagnosed by a psychiatrist.

#### 2) A questionnaire of flood related variables

including height of incoming flood to the house, percentage of flood damage to property, compensation for flood damage, access to health care services after the flood (limited or not limited), and temporary stay in the camp after the flood.

#### 3) Impact of event scale-revised (IES-R)

The IES-R is a 22-item scale in which respondents indicate their experiences over the past seven days using a response scale ranging from “never” (zero) to “very high” (four) [18]. This is composed of eight items related to avoidance symptoms, another eight items on intrusion and six items about hyperarousal symptoms. The range of scores from zero to 88 can be obtained from this scale, so that the higher the score, the more unfavorable the situation. Any individual with a score of 33 or more on the IES-R was defined as a case of PTSD; with a sensitivity of 91% and a specificity of 82% [19]. Panaghi and colleagues have reported acceptable validity and reliability of the Persian version of the scale [20].

### Ethical considerations

Participants provided written informed consent, acknowledging the voluntary nature of their participation and the option to withdraw without consequences. The consent process covered confidentiality and the anonymized publication of results. The project received approval from the ethics committee of the Iran University of Medical Sciences (#IR.IUMS.REC.1398.718), adhering to the principles of the Declaration of Helsinki.

### Statistical analysis

We analyzed the data using STATA version 11 (Stata Corporation, College Station, TX, USA). Continuous variables were represented by the mean and standard deviation or weighted standard error, whereas categorical variables were described using frequency and percentage. Principal component analysis was employed to estimate wealth index and level of damage to assets, considering ownership of assets and the percentage of flood damage to them, respectively [21]. In order to account for the multistage sampling method and non-response adjustment in the analysis, the “svyset” command was employed, introducing the provinces and clusters into the dataset. Sampling weights were computed to adjust each respondent’s representation to encompass other individuals within the study provinces. The post-stratification weights were determined by comparing the proportion of individuals within each stratum from the National Census 2016 to the corresponding proportion observed in the sample. These weights were calculated considering various factors such as age groups (divided into 5 categories), gender, and the 6 counties within the study provinces, resulting in the generation of 60 post-stratification weights. The final weights were determined by multiplying the inverse probability of unit selection into the sample with the post-stratification weights. Statistical tests, including Chi-square, t-test, one-way analysis of variance with Tukey post hoc, and logistic regression analysis, were employed to examine the associations between variables. In the logistic regression analysis, the Hosmer-Lemeshow method was used for modeling [22]. Variables that exhibited a *p*-value below 0.2 in the univariable models were included in the multiple model. Odds ratios, along with their corresponding 95% confidence intervals, were reported to assess the association between the variables and the outcomes. To ensure model validity, potential multicollinearity and interactions were assessed. The fitness of the models was evaluated using the Hosmer-Lemeshow goodness-of-fit test, specifically designed for complex survey data [23]. A *p* < 0.05 was used to determine statistical significance.

## Results

Out of 2,305 people who were approached for surveys, 1,671 (72.5%) flood-affected adults from Golestan (560 of 786), Lorestan (558 of 780) and Khuzestan (553 of 739) provinces participated in the study, and their data were analyzed. The non-response group had a slightly higher mean age (38.9 vs. 36.8 years old) and included a higher percentage of men (81.4% vs. 19.2%). The mean age of participants was 36.8 ± 12.1 years and the minimum and maximum ages were 16 and 85 years, respectively. The majority of participants were housewives, married, illiterate or with primary education, and from rural areas. In regard to flood-related characteristics, 31.5% of participants reported that they temporarily lived in the camps after the flood and for 27%, the height of the flood entering the house was more than one meter. More than 42% of the participants suffered high damage to their assets by the flood and in 74% of the cases, the damage was not compensated. Table 1 shows the demographic and flood-related characteristics of the participants by province.

**Fig. 1:**
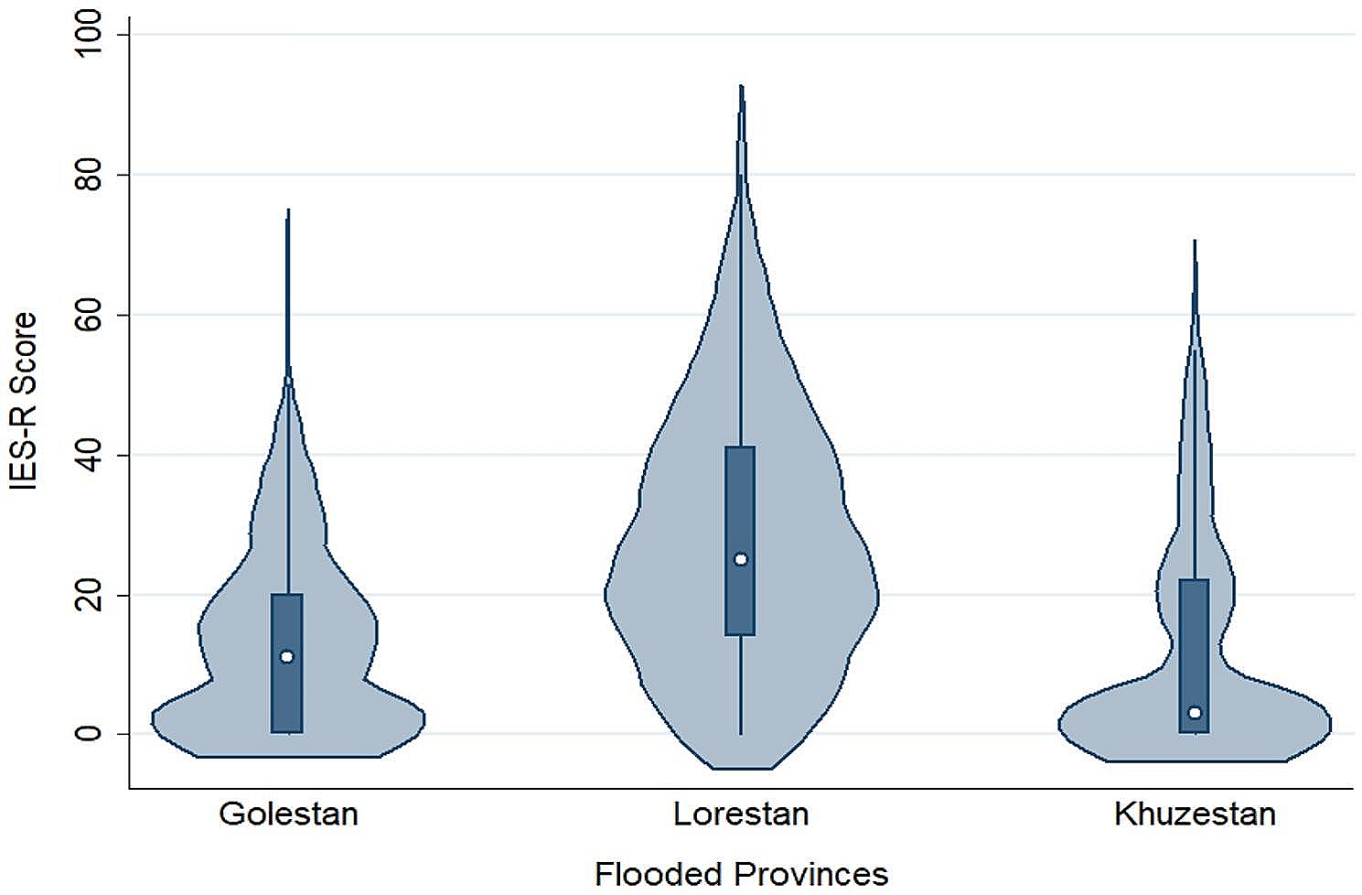
Violin plot for the distribution of IES-R scores in the flooded provinces

The prevalence of PTSD in the participants was 24.8% (CI 95%: 20.7–28.8%). PTSD was more prevalent in female (28.0%, CI 95%: 24.3–31.6%) than male participants (21.6%, CI 95%: 15.0-28.2%), but the difference was not statistically significant (*P* = 0.094). Table 2 shows the prevalence of PTSD in the studied provinces. The prevalence of PTSD was significantly higher in flood victims of Lorestan province (39.7%, *P* < 0.001). The distribution of IES-R score in the flooded provinces can be found in Fig. 1, which shows higher scores in Lorestan province. The highest prevalence of PTSD was observed in participants with a history of mental disorders (42%), followed by people who were exposed to the flood with a height of more than one meter and suffered high damage to their assets (40.7 and 37.8% respectively).

Determinants of PTSD, based on a multiple logistic regression model, were unemployment (adjusted odds ratio (AOR) = 3.53, CI 95%: 1.38-9.00), primary (AOR = 2.44, CI 95%: 1.10–5.41), or high school (AOR = 2.35, CI 95%: 1.25–4.40) education (vs. university), a history of mental disorders (AOR = 2.36, CI 95%: 1.22–4.58), high damage to assets (AOR = 2.29, CI 95%: 1.40–3.75), limited access to health care services after the flood (AOR = 1.95, CI 95%: 1.20–3.19), not receiving compensation for flood damage (AOR = 1.94, CI 95%: 1.01–3.83), high wealth index (AOR = 1.90, CI 95%: 1.23–2.93), and flooded house with a height of more than one meter (AOR = 1.66, CI 95%: 1.02–2.76) (Tables 3 and 4).

**Table 1:** Distribution of demographic and flood related characteristics of the participants and prevalence of PTSD by province

Variable		Golestan *N* = 560		Lorestan *N* = 558		Khuzestan *N* = 553	
		N	Prevalence of PTSD (%)	N	Prevalence of PTSD (%)	N	Prevalence of PTSD (%)
**Age**	16–25 year	102	9.2	59	27.5	132	12.8
	26–35 year	188	7.7	158	42.1	210	11.2
	36–45 year	174	13.6	181	45.7	122	12.2
	46–55 year	72	13.5	90	32.4	52	13.4
	56 ≥ year	24	14.0	70	49.4	37	16.0
**Gender**	Male	69	9.1	78	34.9	174	10.8
	Female	491	12.8	480	44.6	379	14.5
**Marital Status**	Married	474	11.5	444	42.6	462	13.6
	Single	72	11.3	81	29.1	73	7.0
	Divorced or widowed	14	1.5	33	48.7	18	19.9
**Education**	Illiterate or primary	220	17.0	189	52.3	380	15.3
	Secondary or high school	283	9.0	270	39.9	160	8.1
	University	57	4.2	99	25.5	13	5.3
**Employment status**	Employed	54	9.2	42	17.4	84	11.9
	Housewife	427	12.5	400	47.8	356	12.4
	Unemployed	28	12.0	72	45.4	82	17.1
	Other	51	9.3	44	32.4	31	5.6
**Residence**	Urban	384	11.8	255	29.7	0	0.0
	Rural	176	8.5	303	48.5	553	12.6
**Wealth index**	Low or middle	394	9.5	209	39.8	512	13.2
	High	166	13.7	349	39.6	41	5.7
**History of mental disorders**	No	505	10.0	524	38.2	527	11.1
	Yes	55	21.9	34	70.1	26	46.5
**Height of incoming flood to house**	Up to 1 m	497	10.0	290	32.9	486	13.1
	More than 1 m	63	17.5	268	48.6	67	9.1
**Damage to assets**	Low or Moderate	455	7.4	201	31.5	456	11.1
	High	105	21.6	357	44.3	97	20.6
**Compensation for flood damage**	Yes	172	5.8	118	23.0	162	1.0
	No	388	12.8	440	44.7	391	18.3
**Living in camp after the flood**	No	471	11.5	272	33.5	343	15.9
	Yes	89	8.0	286	47.5	210	6.6
**Access to health care services after flood**	Not limited access	514	9.4	369	39.0	395	10.8
	Limited access	46	18.6	189	43.6	158	19.2

**Table 2:** Prevalence of Post-traumatic Stress Disorder in the participants by province

Variable		Golestan province	Lorestan province	Khuzestan province	Total	*P*-value
PTSD based on IES-R	Total score Mean ± SE	13.0 ± 1.0	28.4 ± 1.4	11.4 ± 1.6	20.1 ± 0.8	< 0.001
	Prevalence % (CI 95%)	11.0 (6.1–15.8)	39.7 (32.7–46.8)	12.7 (7.0-18.3)	24.8 (20.7–28.8)	

PTSD = Post-traumatic Stress Disorder, IES-R = Impact of Event Scale-Revised, SE = Weighted Standard Error

**Table 3:** Prevalence of and demographic factors associated with post-traumatic stress disorder in the participants

Variable		N (Weighted %)	Post-traumatic Stress Disorder ^a^		
			*P* %	COR (95% CI)	AOR (95% CI)**
Age (per ten years)		-	-	1.14 (1.01–1.29)*	1.14 (0.96–1.36)
Gender	Male	321 (50.3)	21.6	1	1
	Female	1,350 (49.7)	28.0	1.41 (0.94–2.10)	1.43 (0.84–2.43)
Marital Status	Married	1,380 (75.0)	25.7	1	-
	Single	226 (20.3)	20.6	0.75 (0.44–1.27)	-
	Divorced or widowed	65 (4.7)	27.5	1.09 (0.52–2.31)	-
Education	University	169 (16.4)	17.7	1	1
	Secondary or high school	713 (50.7)	24.5	1.51 (0.82–2.79)	2.35 (1.25–4.40)*
	Illiterate or primary	789 (32.9)	28.7	1.88 (1.02–3.44)*	2.44 (1.10–5.41)*
Employment status	Employed	180 (21.6)	12.4	1	1
	Housewife	1,183 (42.0)	28.0	2.75 (1.26–5.98)*	2.08 (0.83–5.21)
	Unemployed	182 (20.7)	34.0	3.64 (1.41–9.39)*	3.53 (1.38-9.00)*
	Other	126 (15.7)	21.1	1.89 (0.67–5.36)	2.56 (0.82–7.97)
Residence	Urban	639 (51.8)	19.5	1	1
	Rural	1,032 (48.2)	30.4	1.81 (1.13–2.90)*	1.30 (0.77–218)
Wealth index	Low or middle	1,115 (54.3)	19.3	1	1
	High	556 (45.7)	31.3	1.91 (1.31–2.79)*	1.90 (1.23–2.93)*
History of mental disorders	No	1,556 (93.8)	23.6	1	1
	Yes	115 (6.2)	42.0	2.34 (1.24–4.42)*	2.36 (1.22–4.58)*

*P-value < 0.05, P = Prevalence, COR = Crude Odds Ratio, CI = Confidence Interval

**AOR = Adjusted Odds Ratio for Height of incoming flood to house, Damage to assets, Compensation for flood damage, Living in camp after the flood and Access to health care services after the flood

^a.^*P*-value of the Hosmer-Lemeshow test = 0.574, showed no evidence of lack of fit.

**Table 4:** Prevalence of and flood related factors associated with Post-traumatic Stress Disorder in the study participants

Variable		N (Weighted %)	Post-traumatic Stress Disorder ^a^		
			*P* %	COR (95% CI)	AOR** (95% CI)
Height of incoming flood to house	Up to 1 m	1,273 (73.0)	18.9	1	1
	More than 1 m	398 (27.0)	40.7	2.95 (1.88–4.62)*	1.66 (1.02–2.76)*
Damage to assets	Low or Moderate	1,112 (57.6)	15.2	1	1
	High	559 (42.4)	37.8	3.40 (2.20–5.25)*	2.29 (1.40–3.75)*
Compensation for flood damage	Yes	452 (25.6)	12.2	1	1
	No	1,219 (74.4)	29.1	2.94 (1.58–5.48)*	1.94 (1.01–3.83)*
Living in camp after the flood	No	1,086 (68.5)	21.0	1	1
	Yes	585 (31.5)	34.0	2.00 (1.30–3.09)*	1.00 (0.61–1.63)
Access to health care services after the flood	Not limited access	1,278 (75.0)	21.3	1	1
	Limited access	393 (25.0)	36.2	2.10 (1.34–3.29)*	1.95 (1.20–3.19)*

*P-value < 0.05, P = Prevalence, COR = Crude Odds Ratio, CI = Confidence Interval

**AOR = Adjusted Odds Ratio for Age, Gender, Education, Employment status, Residence, Wealth index and History of mental disorders

^a.^Refer to Table 3 footnote.

## Discussion

The prevalence of post-traumatic stress disorder (PTSD) approximately 5 to 6 months after the massive flood of 2019 in Iran was estimated to be around 25%, with the highest prevalence observed in Lorestan province at 40%. This prevalence is near the average prevalence of PTSD in studies of various countries (29.5%), as reported by a systematic review and meta-analysis conducted by Golitaleb et al. in 2022 [24]. According to this review, the sole study concerning floods in Iran between 2015 and 2021 was the investigation conducted by Seyedin et al. [16]. This study examined the impact of the 2012 flood in Behshahr and Neka (Mazandaran province) with a random sample of 400 individuals, three months after the flood, using the PTSS-10 questionnaire and estimated the PTSD prevalence as 64%. A recently published study by Bastami et al. [17] reported a prevalence of PTSD one year after the 2019 flood in Lorestan province as 12.8%, using the Post-Traumatic Stress Disorder Checklist. The prevalence of PTSD has been reported in various populations in Iran, including the general population (2.1%) [25], earthquake victims (51.9% in Bam) [26], veterans, combatants, and freed soldiers (27.8%), and health system workers during the COVID era (14.6%) [27].

The high prevalence of PTSD among 2019 flood victims in Iran, particularly in Lorestan province, can be attributed to several factors. Firstly, the country has a pre-existing high prevalence of psychiatric disorders (23.6%), creating a baseline susceptibility [25]. Recent studies also indicate a rising trend in mental disorders, contributing to an increased burden [28]. Urban settings experience a growing frequency of stress, potentially amplifying vulnerability to PTSD [29, 30]. Economic issues and social factors further exacerbate mental health challenges [31, 32], highlighting the need for comprehensive strategies to address the impact of natural disasters on mental well-being in Iran.

The prevalence of PTSD in the present study was higher in women (28%), but there was no significant difference between two genders. Similarly, in a study on Malaysian flood victims, the higher prevalence of PTSD in women was not significant [33]. In the 2012 flood study in Mazandaran province [16], the PTSD score was significantly higher in men, but in the majority of studies, the female gender was significantly associated with PTSD [17, 34]. Differences in the characteristics and severity of floods, time interval, the demographic composition and context of the studied populations, and the methodologies employed contribute to these variations.

The highest prevalence of PTSD was seen in Lorestan province. Given that the intensity of the flood in Lorestan province was more than the other two provinces, this finding is consistent with the results that show a direct relationship between the intensity of the flood and the prevalence of PTSD [10, 35]. In the present study, the height of the water inundation was measured as one of the indicators of flood severity and it was correlated with the diagnosis of PTSD. The latter finding is consistent with the study of Waite et al. [10]. Several studies have emphasized that flood victims become more vulnerable when confronted with increasingly intense floods [34–36].

According to the present study, unemployment and low education were found to be associated with PTSD. The association of PTSD with low education is consistent with the findings of the assessment of Chinese flood victims in 2000 [37]. Higher education levels may enhance individuals’ knowledge, coping skills, and access to resources, potentially reducing the risk of developing PTSD [38, 39]. A study of PTSD in rural areas of Australia six months after river flooding revealed that socioeconomically marginalized people are more likely to be flooded and forced to leave their homes, and the risk of mental health and PTSD are also higher for cases of house, occupation, or farm inundation [13]. A study of Chinese flood victims also demonstrated that low level of social support is related to PTSD, 17 years after the flood [40] and people with weak social support are less likely to recover from this disorder after 13–14 years [41].

Considering the association between unemployment and PTSD, it is important to explore the possible causal relationship between the two variables. On one hand, the experience of flood trauma and subsequent job loss may contribute to the development of PTSD [42]. On the other hand, it is also plausible to consider that individuals may already have PTSD as a result of various factors following a disaster, which in turn could lead to unemployment [43]. Given the cross-sectional nature of the study and the potential for reverse causality, caution should be exercised when interpreting the observed relationships between unemployment and PTSD.

One of the consequences of the flood was the reduction of access to healthcare services, and this factor had a significant correlation with PTSD. A study by Waite et al. [10] also found in England that impaired access to health and social care, as well as education and work, was associated with PTSD.

In contrast to previous studies [34], the present study found a higher prevalence of PTSD among individuals with a high wealth index. This unexpected finding may be attributed to the fact that individuals who had greater assets prior to the flood may have experienced greater difficulties in coping with the aftermath of the disaster. The loss of their assets without the prospect of compensation could contribute to their heightened distress and lack of hope for recovery.

The results indicated that individuals with a history of mental disorders are more susceptible to developing PTSD following a traumatic event such as a flood. This finding underscores the importance of considering mental health history when prioritizing services and conducting PTSD screening for flood victims [34].

Apart from the obvious need to address the socio-economic situation of flood victims and plan to prevent future incidents, it is recommended to screen high-risk and vulnerable groups based on the factors obtained from the present study and prioritize resources for managing the mental health of this population. Considering the prolongation of PTSD after the huge flood, periodic screening of the affected population and the preparation of appropriate therapeutic and supportive interventions are recommended.

### Limitations

One limitation is the lack of use of a diagnostic interview, which is often considered the gold standard for PTSD diagnosis. However, in studies with large sample sizes, it is usually neither cost-effective nor feasible to use this method. Nevertheless, the use of self-report questionnaires is inherently more biased than the diagnostic interview. We addressed this concern by employing a standardized and validated questionnaire, clarifying the study goals, and ensuring the participants about the confidentiality of the findings.

The study acknowledges a potential source of error in the data collection process attributed to the practices of 18 data collectors. To address this issue, they received training based on an executive protocol. Despite these efforts, the study recognizes the inherent subjectivity in questioning as a limitation.

The flood and the destruction of some houses caused the displacement of a number of people and their withdrawal from the sampling process. Paying attention to the fact that the prevalence of PTSD in this group of people is usually higher, it can lead to an underestimation of this disorder. When generalizing the results, it is essential to take into account the study setting and the characteristics of the participants.

## Conclusion

In conclusion, our study highlights a concerning prevalence of PTSD, particularly in Lorestan province, among adults affected by severe flooding in Iran. Independent determinants of PTSD include unemployment, lower education, a psychiatric history, extensive property damage, limited post-flood healthcare access, lack of compensation, and greater flood exposure. We recommend adopting an inclusive screening approach for high-risk groups based on identified factors, prioritizing resources for mental health management, and developing appropriate therapeutic and supportive interventions.

## Data Availability

The data that support the findings of this study are available from the corresponding author, upon reasonable request.

## Abbreviations

PTSD: Post-traumatic Stress Disorder
IES-R: Impact of Event Scale-Revised
CI: Confidence Interval
AOR: Adjusted Odds Ratio
COR: Crude Odds Ratio
